# Current News

**Published:** 1894-08

**Authors:** 


### Current News.

### WOMAN'S DENTAL ASSOCIATION OF THE UNITED STATES.

A regular monthly meeting was held May 5, 1894, at 1602 Arch Street, Philadelphia, Dr. Anna T. Focht, President, in the chair.

Dr. James Truman gave an interesting and instructive talk on Dental Pathology.

A regular monthly meeting was held June 2, 1894, at 1300 Arch Street, Philadelphia, the President, Dr. Anna T. Focht, in the chair.

Dr. Bertha Jarrett read a paper on "Local Anæsthesia." The discussions brought out especial denouncements of the "Local Anæsthetic Man" who is working such havoc.

The next meeting will be held September 1, 1894, at Dr. Focht's office, Broad and Columbia Avenue.

EMILY W. WYETH,
*Recording Secretary.*

3920 FAIRMOUNT AVENUE.

---

### RECENT PATENTS.

A LIST of recent patents reported specially for the INTERNATIONAL DENTAL JOURNAL:

514,073.—Angle Attachment for Dental Handpieces. Frank K. Hesse, Boston, Mass., assignor to Codman & Shurtleff, same place. Filed November 2, 1892.

514,074.—Angle Attachment for Dental Handpieces. Frank K. Hesse, Boston, Mass., assignor to Codman & Shurtleff, same place. Filed March 11, 1893.

514,201.—Mechanical Dentistry. Lucius Robertson, Cincinnati, Ohio. Filed March 3, 1893.

514,289.—Dental Clamp. Joseph M. Strout, Portland, Me. Filed April 8, 1893.

514,882.—Dental Mandrel. Walter S. Elliott, Sag Harbor, N. Y., assignor of one-half to James W. Ivory, Philadelphia, Pa. Filed April 24, 1893.

514,941.—Dental Tool. Frederick A. Kotts, Manchester, Mich. Filed July 15, 1893.

515,126.—Dental-Engine Mallet. Frank M. McCarty, Shelbyville, Ind. Filed April 1, 1893.

515,400.—Artificial Tooth. Hardy B. Harrell, Gainesville, Tex. Filed December 29, 1892.

516,310.—Dental Impression-Cup. Henry L. Knight, Le Roy, Minn., and Hobert E. Duncan, West Superior, Wis. Filed June 15, 1893.

516,465.—Dental Engine. Frederick H. Berry, Milwaukee, Wis. Filed June 21, 1893.

516,529.—Dental Articulator. Florian E. Hansen, Minneapolis, Minn. Filed December 2, 1893.

516,700.—Dental Vulcanizer. Charles A. Davis, Pasadena, Cal. Filed December 13, 1893.

517,151.—Process of Manufacturing Dental Plates. George A. Tompkins, Cortland, N. Y. Filed February 23, 1892.

517,218.—Dental Engine-Head. Andrew J. Harris, Chicago, Ill. Filed October 22, 1892.

517,248.—Angle Attachment for Dental Handpieces. Rufus G. Stanbrough, New York, N. Y. Filed December 2, 1892.

517,249.—Angle attachment for Dental Handpieces. Rufus G. Stansbrough, New York, N. Y. Filed February 20, 1893.

518,175.—Dental Handpiece. Stephen H. Brooks, New York, N. Y., assignor to Edward A. Peirce, same place, and J. Otis Cox, Brooklyn, N. Y. Filed October 31, 1893.

518,701.—Dental Disk-Carrier. George B. Richmond, Lansing, Mich. Filed September 15, 1893.

518,877.—Dental Chair. John Hood and Stephen H. Reynolds, Boston, Mass. Filed January 27, 1893.

519,109.—Dental Chair. Thomas H. Gardner and Edward Gardner, Manchester, England. Filed December 6, 1892. Patented in England.

519,110.—Retaining or Lowering Apparatus. Thomas H. Gardner and Edward Gardner, Manchester, England. Filed November 20, 1893. Patented in England and France.

519,756.—Dental Chair. Arthur W. Browne, Prince's Bay, N. Y., assignor to the S. S. White Dental Manufacturing Company, Philadelphia, Pa. Filed February 5, 1894.

519,757.—Dental Chair. Arthur W. Browne, Prince's Bay, N. Y., assignor to the S. S. White Dental Manufacturing Company, Philadelphia, Pa. Filed February 6, 1894.

519,758.—Dental Chair. Arthur W. Browne, Prince's Bay, N. Y., assignor to the S. S. White Dental Manufacturing Company, Philadelphia, Pa. Filed February 6, 1894.

519,799.—Light-Concentrator for Dentists, Surgeons, or others. William H. Thrift, Dubuque, Iowa. Filed September 5, 1893.

519,845.—Gas-Regulator. Theodore G. Lewis, Buffalo, N. Y., assignor to the Buffalo Dental Manufacturing Company, same place. Filed February 15, 1894.

519,883.—Dental Chair. Arthur W. Browne, Prince's Bay, N. Y., assignor to the S. S. White Dental Manufacturing Company, Philadelphia, Pa. Filed February 5, 1894.

519,884.—Dental Chair. Arthur W. Browne, Prince's Bay, N. Y., assignor to the S. S. White Dental Manufacturing Company, Philadelphia, Pa. Filed February 5, 1894.

520,593.—Tooth-Brush. Otto F. Hager, Buffalo, N. Y. Filed February 10, 1894.

520,867.—Dental Engine. Rufus G. Stanbrough, New York, N. Y. Filed December 2, 1892.

520,947.—Dental Chair. Arthur W. Browne, Prince's Bay, N. Y., assignor to the S. S. White Dental Manufacturing Company, Philadelphia, Pa. Filed December 27, 1893.

520,948.—Dental Chair. Arthur W. Browne, Prince's Bay, N. Y., assignor to the S. S. White Dental Manufacturing Company, Philadelphia, Pa. Filed February 5, 1894.

521,138.—Electric Motor for Dental Engines. Walter A. Crowdus, Chicago, Ill., assignor to the Turney Electric Manufacturing Company, same place. Filed October 22, 1892.

Trade-Marks.—24,640.—Dentifrice. Luis J. de Carballo, New York, N. Y. Filed April 2, 1894. Essential feature the word "Eulalia."

24,846.—Dental Preparations. Joaquin A. Bermudez, New Orleans, La. Filed May 14, 1894. Essential feature, the word "Cameo."

Design.—23,189.—Dental Cabinet. William G. Hullhorst, Toledo, Ohio, assignor to the Ransom & Randolph Company, same place. Filed March 10, 1894. Term of patent, seven years.

### LIST OF EXPIRED PATENTS.

187,573.—Dental Chairs. S. S. White, Philadelphia, Pa. Filed January 16, 1877.

188,329.—Dental and Barbers' Chairs. George W. Archer, Rochester, N. Y. Filed October 17, 1876.

189,249.—Dental Pluggers. K. L. Mills, Kansas City, Mo. Filed February 22, 1877.

189,409.—Dental Engines. B. M. Wilkerson, Baltimore, Md. Filed February 13, 1875.

189,526.—Dental Engines. G. W. Tripp, Auburn, N. Y., assignor to S. S. White, Philadelphia, Pa.

189,735.—Dental-Foil Condensers. John Hood and S. H. Reynolds, Boston, Mass. Filed October 25, 1876.

190,115.—Dental Tools. S. Babcock and C. E. Mason, Springfield, Ill. Filed November 29, 1876.

Reissue.—7670.—Dental Engines. Charles M. Curtis, Philadelphia, Pa., assignor, by mesne assignments, to M. M. Johnston, New York City. Patent No. 138,318, dated April 29, 1873. Filed April 23, 1877.

